# A neurologist’s rhombencephalitis after comirnaty vaccination. A change of perspective

**DOI:** 10.1186/s42466-021-00156-7

**Published:** 2021-11-08

**Authors:** Alexander Walter, Markus Kraemer

**Affiliations:** 1 Department of Psychiatry, Kliniken Essen-Mitte/ Huyssenstift, Essen, Germany; 2 Department of Neurology, Alfried-Krupp-Krankenhaus Essen, Essen, Germany; 3 Department of Neurology, Medical Faculty, J`Heinrich Heine University Duesseldorf, Duesseldorf, Germany

## Abstract

Rhombencephalitis is an orphan disease of multiple causes that may manifest with facial palsy, limb ataxia and reduced consciousness. Up to now it is described after COVID-19 infection and in this (personal) case was found up to 8 weeks after Comirnaty vaccination. So far, we do not fully understand the pathophysiological characteristics of encephalitis associated with SARS-CoV-2. In rare cases, vaccination may cause an immunological reaction and delayed inflammation, the consequences of which we have not yet deciphered. Rhombencephalitis should be considered as a rare potential mRNA-associated vaccination side effect.

**Supplementary Information:**

The online version contains supplementary material available at 10.1186/s42466-021-00156-7.

Dear Ladies and Gentlemen,

I was 30 years old when I realized that something was wrong. As a medical doctor, I have worked at neurology department for more than four years. I received the first dose of Comirnaty (Pfizer/ Biontech) vaccine on 19 January and the second dose on ninth of February 2021.

In April, I first noticed a generalized malaise, moderate headache and taste disorders. Within 2 weeks, I developed a facial paralysis on the left side, discreet paralysis of the hypoglossal nerve on the right side and a massive ataxia of all extremities (Table 1).

**Table 1:** Development of neurological symptoms

Point in time	Symptoms and clinical findings
April 2021	1. Generalized malaise and moderate headache
April 2021 (2 days later)	2. Taste disorders, facial paralysis on the left side
May 2021 (1 week later)	3. Progressive gait disturbance caused by a massive ataxia of all extremities
May 2021 (a few days later)	4. Paralysis of the hypoglossal nerve on the right side

The MRI of the brain showed a weak FLAIR hyperintensity of the brainstem, mesencephalon and cerebellar around the fourth ventricle without contrast enhancement. MRI of cervical and thoracic spine were without pathological findings.

Lumbar puncture showed an inflammation of 10 cells/µl (normal range ≤ 5 cells/µl). Neurography and a second lumbar puncture later on were normal. CSF analysis revealed 2 cells/µl, normal protein (401 mg/l) and glucose (72 mg/dl). Bacterial culture and other tests including HSV, VZV, EBV, CMV, borreliosis and sarcoidosis were negative. Genetic testing for HLA-B-51 was negative. Strikingly, no positive antineuronal, paraneoplastic, or antiganglioside antibodies were present in serum or CSF. Oligoclonal bands were negative in both punctures, and there was no albumin cytologic dissociation. Full blood tests including ANA, ANCA, Syphilis, anti-MOG, Aquaporin-4 antibodies and the HIV test were negative.

After careful evaluation by neurologic experts, an autoimmune rhombencephalitis was diagnosed. As rhombencephalitis has been described to be associated with COVID-19 infection [1–3], it was hypothesized that in my case, Comirnaty vaccination was the immunological trigger. This is the first report about an autoimmune rhombencephalitis after Comirnaty vaccination whereas e.g. Guillian-Barré syndrome, which is pathophysiological related, was described quite often after that vaccine [4].

A high dose intravenous cortisone therapy with 1000 mg methylprednisolone per day for a total of 5 days was initiated. Thereafter, oral corticosteroids were tapered within two weeks starting with 80 mg prednisolone per day.

As a result, the neurological symptoms, especially the ataxia, rapidly decreased. I was referred to an acute rehabilitation facility where most symptoms improved significantly within a few weeks.

Why did I write this article? What do we learn from it?

Firstly, I think it is an interesting and rare, atypical case of rhombencephalitis. I have learned a lot about the very low prevalence of my disease, the (unclear) prognosis, the treatment options, the possible complications, and the possible connection with COVID-19 vaccination. Even though the time between vaccination and the appearance of symptoms seems to be rather long, we did not find any other trigger for this orphan disease.

Secondly, it showed me how quickly one can fall ill and that there is no guarantee of health.

Thirdly, it was very difficult for me as a neurologist to recognize specific neurological symptoms in myself. It makes a great difference if you examine a patient or if you are the patient. It makes me compliant, cautious, and grateful.

Finally, as a scientist, it has shown me that we do not yet fully understand the pathophysiological characteristics of encephalitis associated with SARS-CoV-2.

Increasing evidence suggests that there is a wide range of complications of SARS-CoV-2 infection, including neurological disorders [5]. Guillain-Barré syndrome, Bickerstaff-Encephalitis, Miller Fisher syndrome, acute necrotizing encephalitis, myelitis, acute disseminated encephalomyelitis (ADEM), and myasthenia gravis have been reported after COVID-19 [1, 6–9]. All reviewed studies demonstrate that neurological manifestations are broad and heterogeneous suggesting different underlying pathogenic processes and pathways [10].

The possible relationship between the COVID-19 infection and these neuroinflammatory diseases would be consistent with a post-infectious immune-mediated mechanism. It is imaginable, that this may also occur after a vaccination.

In literature, we also found some cases with an encephalitis related to a H1N1 vaccination [11], which support our hypothesis.

In addition to that, smell and taste disorders are common with COVID-19. They appear especially in previously healthy, young people [12]. In my case, I also had taste disorders as an early symptom. This could be a supplementary hint for a similar pathophysiological origin (Additional file 1).

Furthermore, we did not find any other causes for this type of encephalitis. We could eliminate other infectious, autoimmune, paraneoplastic and genetic causes by blood testing and MRI imaging.

In conclusion we do not yet know much about complications after vaccination [13]. Currently, there is not much data available [4]. In rare cases, vaccination may cause an immunological reaction and delayed inflammation, the consequences of which we have not yet deciphered. An autoimmune rhombencephalitis may be considered as a rare potential mRNA-associated vaccination side effect [1, 3].

Follow-up studies will be necessary to ascertain the long-term neurological consequences of COVID-19 vaccines.

## Description of the MRI findings

The MRI of the brain shows an axial FLAIR on the right side with a weak hyperintensity cerebellar around the fourth ventricle. On the left side is a sagittal T2 weighted image, which illustrates a bright abnormality of the rhombencephalon. The conspicuous features are marked with a red arrow.

## Supplementary Information

**Additional file 1**. The main results of the MRI of the brain: A abnormality of the rhombencephalon and cerebellum.

## Data Availability

All further datasets or information are available from the corresponding author on reasonable request. The main data analysed during this case are included in this article.
