# Supplementary figures and images for: A neurologist’s rhombencephalitis after comirnaty vaccination. A change of perspective
Source: Neurol Res Pract. 2021 Nov 8;3:56. doi: 10.1186/s42466-021-00156-7 (PMC8572650; doi:10.1186/s42466-021-00156-7)

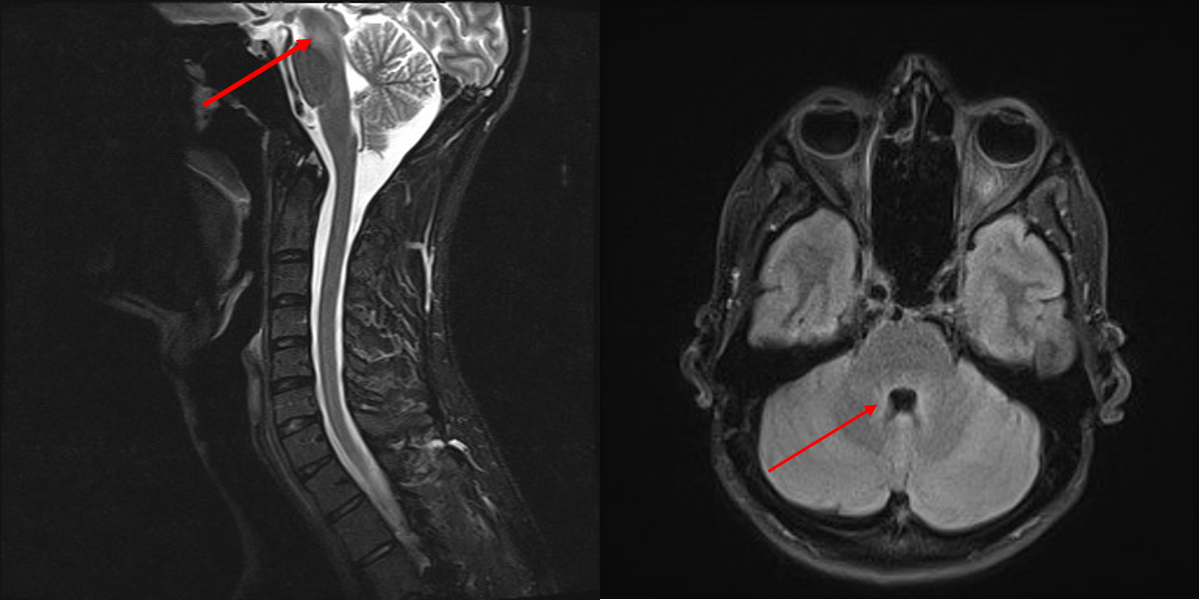

Supplement: Supplementary file 1 — Additional file 1. The main results of the MRI of the brain: A abnormality of the rhombencephalon and cerebellum. [file 42466_2021_156_MOESM1_ESM.tif]
